# Ameliorating aging‐associated cognitive decline by physical exercise: multi‐organ integrative approach (EMBRACE & AMETHYST trials from Slovakia)

**DOI:** 10.1002/alz70860_102754

**Published:** 2025-12-23

**Authors:** Barbara Ukropcová

**Affiliations:** ^1^ Institute of Pathophysiology, Faculty of Medicine, Comenius University, Bratislava, Slovakia; Biomedical Research Center, Slovak Academy of Sciences, Bratislava, Slovakia

## Abstract

Sedentary lifestyle accelerates aging‐associated decline of motor, metabolic and cognitive functions. Deteriorated structural and functional capacity is linked to chronic subclinical inflammation, mitochondrial dysfunction and insulin resistance, operating across multiple tissues, including the brain. These pathomechanisms underlie hippocampal atrophy and cognitive impairment and contribute to development of Alzheimer's and Parkinson's disease.

Regular moderate‐to‐vigorous exercise ameliorates aging‐associated cognitive, motor and metabolic decline. Both strength and aerobic exercise were shown to positively modulate cognition and brain plasticity. Yet, exercise as a readily available physiological modality of prevention and treatment of cognitive decline is relatively scarcely employed.

Highly coordinated nature of a synchronized adaptive response to exercise suggests the key role for systemic mediators, which are collectively termed exerkines. Contracting skeletal muscle was identified as an organ secreting many such bioactive compounds, and is one of the most robust contributor to the systemic pool of exerkines.

Randomized clinical trials EMBRACE and AMETHYST are focused on effects of a relatively short (3 to 9‐month) and long term (≥5 years) supervised aerobic‐strength exercise on cognitive functions, brain morphology, skeletal muscle and systemic metabolism in a well characterized prospectively followed population. Non‐invasive approaches of high field magnetic resonance (MR) imaging and spectroscopy and the capacity to link results of the multimodal & multiorgan MR examination to the whole‐body and muscle metabolic phenotypes as well as to cognitive functions, physical fitness and quality of life and the availability of the prospectively collected samples of biological material (plasma, muscle, cerebrospinal fluid/CSF) provide us with the opportunity to identify effects of exercise and mechanisms of exercise‐induced adaptive response at a systemic, organ and molecular level. We described regulation of bioactive molecules in plasma and CSF in response to exercise and their link to cognitive performance. Importantly, we demonstrated sustainability and efficiency of long‐term training programs in the elderly. The major focus of our work is fully in line with TEIDe's objectives. Participation in TEIDe consortium provides us with the excellent opportunity to generate more robust data and to become a part of network implementing individualized exercise prescription in clinical practice.

Grant support: HORIZON Europe ADDIT‐CE, APVV‐20‐0466, VEGA‐2‐0766, APVV‐23‐0406

